# Patients' and Health Professionals' Experiences of Using Virtual Reality Technology for Upper Limb Training after Stroke: A Qualitative Substudy

**DOI:** 10.1155/2018/4318678

**Published:** 2018-02-08

**Authors:** Hanne Pallesen, Mette Brændstrup Andersen, Gunhild Mo Hansen, Camilla Biering Lundquist, Iris Brunner

**Affiliations:** ^1^Hammel Neurorehabilitation Centre and University Research Clinic, RM, University of Aarhus, Aarhus, Denmark; ^2^Department of Global Public Health and Primary Care, University of Bergen, Bergen, Norway

## Abstract

**Background:**

In recent years, virtual reality (VR) therapy systems for upper limb training after stroke have been increasingly used in clinical practice. Therapy systems employing VR technology can enhance the intensity of training and can also boost patients' motivation by adding a playful element to therapy. However, reports on user experiences are still scarce.

**Methods:**

A qualitative investigation of patients' and therapists' perspectives on VR upper limb training. Semistructured face-to-face interviews were conducted with six patients in the final week of the VR intervention. Therapists participated in two focus group interviews after the completion of the intervention. The interviews were analyzed from a phenomenological perspective emphasizing the participants' perceptions and interpretations.

**Results:**

Five key themes were identified from the patients' perspectives: (i) motivational factors, (ii) engagement, (iii) perceived improvements, (iv) individualization, and (v) device malfunction. The health professionals described the same themes as the patients but less positively, emphasizing negative technical challenges.

**Conclusion:**

Patients and therapists mainly valued the intensive and motivational character of VR training. The playful nature of the training appeared to have a significant influence on the patients' moods and engagement and seemed to promote a “gung-ho” spirit, so they felt that they could perform more repetitions.

## 1. Introduction

Stroke is one of the most frequently occurring diseases in modern society and often leads to lifelong critical disability [1, 2]. Approximately two-thirds of stroke survivors experience motor deficits of the upper limb, resulting in reduced quality of life [3]. Repetition is one of the key factors in regaining motor function after stroke [4, 5]. Studies in animals have shown that at least 400 repetitions are needed to induce plastic changes in the brain [4].

Over the last 10 years, virtual reality (VR) technology has been introduced into neurorehabilitation, in particular with the intention of facilitating motor function recovery by way of many repetitions [6]. Novel VR rehabilitation systems increase intensity and seem to offer challenging and motivating tasks [7]. Upper limb VR training provides a higher degree of activity, compared with conventional training, for severely affected subacute patients after stroke [8]. There is not yet much data on the use of VR systems for rehabilitation and/or commercial gaming devices in clinical practice for upper limb rehabilitation after stroke [9]. A British survey concluded that commercial gaming consoles are used by almost a fifth of therapists. Gaming was reported to be enjoyable, but therapists described barriers related to time, space, and cost [10]. Few studies have focused on patients' and therapists' perceptions of upper limb VR training [11, 12]. Participants with chronic stroke reported that the upper limb VR training was motivational; however, they expressed frustrations about technical challenges [12]. According to another study, which concentrated on the therapist perspective, VR was perceived as a useful additional treatment tool to complement conventional methods. However, it was emphasized that VR could not replace the therapist's clinical reasoning or their social interaction with the patients [11].

A growing number of studies suggest that VR for upper limb training could be beneficial in the chronic phase [13]. However, only a few minor studies have enrolled patients in the acute and subacute phases. Furthermore, none have focused on the subacute stroke patient's experiences of VR upper limb training. Therefore, a qualitative substudy was conducted as part of the Virtual Reality for Upper Extremity after Stroke (VIRTUES) trial. This trial is registered with ClinicalTrials.gov NCT02079103. This randomized controlled multicentre trial was performed at five rehabilitation hospitals in three countries to examine the benefit of VR technology on upper limb motor recovery after stroke [14]. Over a period of four weeks, 120 patients were offered additional VR arm training in 4-5 training sessions a week, each of 45–60 minutes' duration, led by a physiotherapist or an occupational therapist.

### 1.1. The VR System

The YouGrabber system (YouRehab Ltd., Switzerland) is an example of a therapeutic VR upper limb training in stroke rehabilitation. The YouGrabber system includes wearable data gloves with sensors and training software with a range of gaming options. The system offers several VR rehabilitation scenarios, providing a graded training programme of goal-oriented reaching and/or grasping exercises. YouGrabber (YG) was chosen because its range of therapy modes allows the inclusion of patients with a broad range of arm motor impairments, from mildly to severely reduced function, and because of its moderate costs, compared to other technology-based rehabilitation products [14]. YG offers participants seven different games, giving visual feedback upon attainment for each game (Airplan, Magic Finger, ToyCatching, Catch the Carrot, Tomato Juggling, and Shopping).

The purpose of this article is to explore patients' and therapists' experiences of using a VR rehabilitation system for upper limb training after stroke. The current study focuses on experiences of VR upper limb training in the subacute phase.

## 2. Methods

### 2.1. Study Design

A qualitative approach was chosen, because the aim of the study was to illuminate the perspectives and experiences of the individual stroke patient and therapist using VR arm training in subacute rehabilitation setting in Denmark. A qualitative approach enables the researcher to enter the world of the participants and gain insight into their thoughts and feelings [15]. The interviews were inspired by the phenomenological theoretical perspective and aimed to explore the participants' perceptions, interpretations of their own experiences, and everyday explanations. Stroke individuals were regarded as being experts in their own experiences [16, 17].

As a supplement, a questionnaire survey, addressing satisfaction with the VR training, was conducted among the health professionals and patients involved in the main study. The purpose of this methodological strategy is to use the results of the quantitative data to support the validity of the qualitative findings [18].

The questionnaire was developed for this purpose. All patients in the VR training group were handed out a simple questionnaire after they had completed the intervention. They were asked to rate their satisfaction with VR training, their perceived improvement due to VR, and the extent to which they experienced VR training as motivating. Comments could be added in free text.

### 2.2. Central Concepts

The interrelated concepts of the body control, disability, gaming, and playing became the foundation through which the data were understood and interpreted. The analysis drew upon theories of the lived body [16]. The body (as well as the affected upper limb) can be made the object of training, treatment, and optimization of function, both by the person inhabiting it and by therapists. At the same time, the body (as well as the affected upper limb) is a significant part of the individual's perception, emotional joy, and pleasure as well as discomfort and endurance. The upper limb can be regarded at once as a medium to fulfil one's needs and the connection to the surrounding world and social relationships [16]. The data related to gaming and playing were structured and interpreted in accordance with James Carse's theories of finite and infinite games [19]. Briefly, both finite and infinite games are played in accordance with rules, as agreed upon by the participants. However, the signification of the rules is different for the two types of games. Boundaries in finite games are “rules” within which one must remain when playing a finite game, in contrast with horizons in infinite games, which move with the player and are constantly changing as he or she “plays” [19]. We used the theory of finite games to conceptualize and understand the different experiences and meanings expressed by the stroke individuals and the therapists.

### 2.3. Selection of Participants

The stroke individuals were selected to represent the stroke population, that is, people over the age of 30. In addition, they were selected to cover a range of different levels of upper limb motor impairment, as well as various levels of experience in using technology equipment. This strategy was chosen to get a maximum variation of informants and they were recruited from the last half of the Danish patients enrolled from the main randomized, controlled, single-blind phase III multicentre trial. The level of these experiences was estimated by VIRTUES therapists who led and supported the patients in the YG training sessions. Patients with limited speech ability were not included.

The patients were interviewed between January 2016 and September 2016.

See Table 1.

Three therapists, who led the YG training in the ward for patients with moderate impairment after stroke, participated in the first focus group interview (F1).

In the second focus group interview (F2), four therapists who led the YG training on a ward for patients with moderate-to-severe impairment after stroke participated. See sociodemographic characteristics of the participants in the two focus groups (Table 2).

The occupational therapist and physiotherapist had to fulfil the selection criteria of being experienced in working with YG systems and have conducted a minimum of sixteen YG stroke arm therapy sessions. Focus groups were conducted between September and October 2016.

None of the invited therapists or patients declined to participate or withdrew consent.

### 2.4. The Interviews and Interview Guide

The interview guide was tested for comprehensibility by two former VIRTUES patients before the actual data collection. Small corrections were made. Then semistructured face-to-face interviews were conducted with six patients in the final week of their VR upper limb training. The final week was chosen to ensure that the informants had an appropriate amount of experience, not too affectionate or too bored, and also to make sure that they still had present memory of the training.

One of the interviews was performed as a dyad-interview (patient numbers 2 and 3). These two patients were inpatients on the same ward and had accompanied each other on the YG training. It was therefore considered that interviewing them together could enrich the interview data. The two focus group interviews with therapists were carried out after the intervention had been completed. The interviews were performed by the authors HP, GMH, and CBL.

The interview guide comprised four main categories: (1) general overall impression of YG training, (2) experiences of other rehabilitation technology, (3) experiences of progress in YG training, and (4) the therapists' learning strategies, support, organization, and facilitation of the YG training (see interview guide, Table 3).

Basically, the same interview guide was used for patients and therapists, and the perspective was changed accordingly.

### 2.5. Procedures

The interviews with stroke individuals were conducted on the ward in a separate, small meeting room. The focus group interviews with therapists were also carried out on the ward, in a larger room. The interviews were led by a moderator, the first author (HP), while the third (GMH) and fourth (CBL) authors of this article asked supplementary questions.

Before the interview got underway, the moderator explained the process. The moderator followed a standardized procedure of organizing, conducting, recording, and handling data [15].

The interviews with the stroke individuals took place ahead of the focus group interviews; interesting issues raised by the patients could thereby be elaborated and discussed later in the therapists' interviews.

The data from the two types of interviews were analyzed separately.

### 2.6. Data Analysis

The data analysis was inspired by the phenomenological approaches of Giorgi [20, 21], Zahavi [22], and Kvale and Brinkmann [15] and was carried out using an iterative process motivated by Giorgi's levels of analysis [20]. Pursuing a phenomenological perspective involved the use of the phenomenological notions of “reduction,” “epoché,” and “intentionality,” capturing a reflective position and simultaneously neutralizing a dogmatic attitude, taking into account the fact that preconceptions cannot be avoided or overlooked [22].

The empirical data were transformed into closely related empirical themes. The data preparation and analysis process is described below.

*Data Preparation. *All the data material from the interviews, four individual interviews, one dyad-interview, and two focus group interviews, was transcribed verbatim by a research assistant (MBA). The interview transcript was then read by the researcher, after which the interview was played again and corrections were made to the transcript. Then the interview transcript was transferred into a qualitative research software programme, NVivo10 (QSR International, Doncaster, Victoria, Australia), which was used to store and organize the data during the analysis.

*Analysis Process. *The analysis process was carried out in line with Giorgi's four levels of analysis [20] by the first (HP) and second (MBA) authors.

**Step 1 (read for sense of the whole).**

The transcripts of the interviews were slowly read through to capture the description as a whole, while simultaneously listening attentively to the audio-recorded interviews, ensuring that the transcription was accurate. The analytical process was supported by both a manual (HP) and provisional categorization of each interview, whereupon individual interviews were encoded using the NVivo 10 computer programme (MBA).

**Step 2 (determination of meaning units).**

Subsequently, meaning units and possible thematic patterns were traced and provisional, compatible patterns in the empirical data were manually identified by the authors. Identified meaning units and traces of thematic patterns were also transferred into NVivo 10. In this way, an analytical generalization of the material was carried out, so that knowledge moved from individual to pattern among a group to general dynamics [20].

**Step 3 (transformation of participants' natural attitude expressions into phenomenological, sensitive expressions).**

Meaning units were then organized and data from the individual, duo-interviews, and focus group interviews were gradually transformed into categories that represented a solid general meaning, moving from situated structures to a general structure. In an iterative process of going back to the raw data and simultaneously condensing meaningful structures, still more essential meaning emerged, and the most representative and clarified meaning units were then organized.

**Step 4 (writing the general structure of what was experienced).**

**Figure 1:**
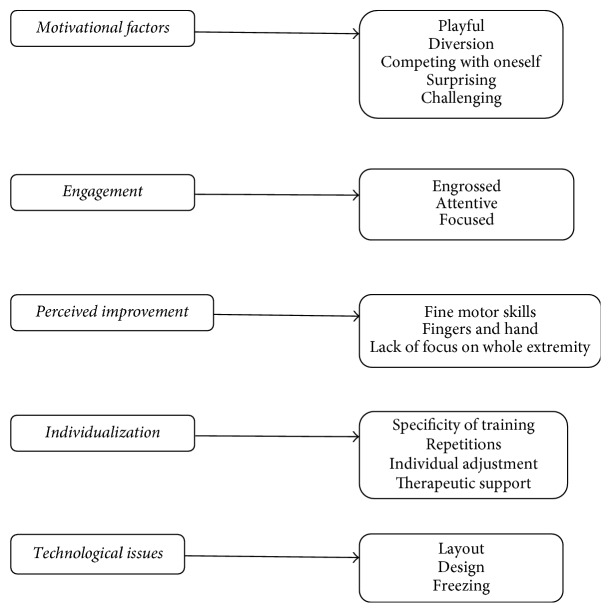
Themes from patients' and therapists' interviews.

**Figure 2:**
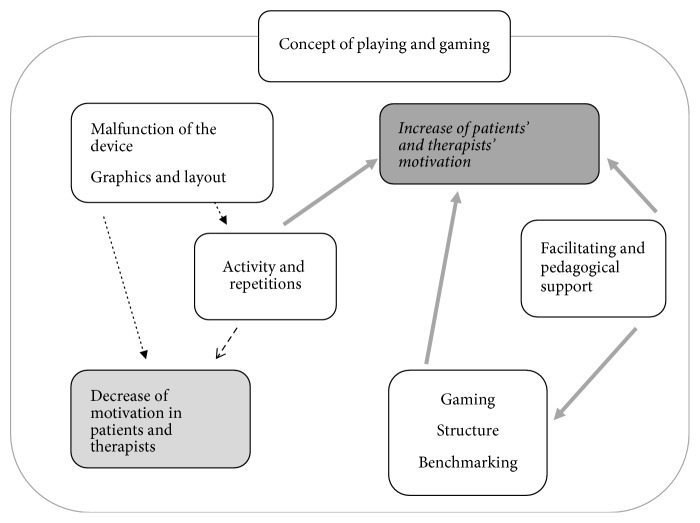
Discussion of the findings.

The empirical data were eventually synthesized and described in a final set of themes and subthemes. The themes responded to the research questions and described a general structure of the experiences as revealed in the data [20] (see Figures 1 and 2). During the whole process of data analysis, the data were communicatively validated by the first and the second authors using the concepts of transferability, credibility, dependability, and confirmability [23]. The final results were discussed and interpreted within the theoretical framework and cogenerated by all authors, who offered their experiences from their fields. Here, methods of triangulation were used to increase the credibility of the study. The results were thereafter deliberated on and were considered to constitute new knowledge.

The questionnaire survey was analyzed using descriptive statistics in SPSS 22 (Armonk, NY: IBM Corp).

## 3. Ethical Considerations

Ethical approval for this study was granted by the Danish Data Protection Agency 2007-58-0010 (study number 636) and the study was completed in accordance with the Helsinki Declaration 2008. The participation of stroke individuals and therapists was voluntary and withdrawal was possible at any time. Anonymity was preserved; thus, the names and identifiable places or situations related to the participants have been changed. Oral and written consent to participate were obtained.

## 4. Findings

In total, five interviews with six patients (four individual interviews and one dyad-interview) and two focus groups with seven occupational therapists and physiotherapists were conducted. For details, see Tables 2 and 3. The duration of each interview was between 22 and 40 minutes. Based on the information from the transcripts, encoding, and categorization, five main themes finally emerged: (i) motivational factors, (ii) engagement, (iii) perceived improvements, (iv) individualization of arm training, and (v) frustrations about device malfunctions. These five themes illuminate the patients' and therapists' experiences of using the YG system for rehabilitation of upper limb training (see Figure 1).

Finally, (vi) general impression of satisfaction with VR training is added to the findings. This theme emerged from questionnaires.

## 5. Motivational Factors

The patients highlighted a number of factors that influenced their motivation during the VR training:

I thought it was fun. (All patients)

They cherished the playful nature of the treatment and the games. One of the patients expressed in an indirect way how much she enjoyed the session by saying the following:

My problem, it's that they say, now it's over. (Patient number (Pn) 5)

An older male patient who had never been engaged in technology or gaming expressed his joy differently:

Well, it was, you know, a break. It was completely different. (Pn 4)

Several of the patients experienced the sessions of YG arm training as a break and diversion from their daily routine.

They reported that they trained unconventionally and that the sessions were not quite planned and the game offered unexpected surprises:

Something very different. I didn't know, when I went down (to the training), what we were going to do. I don't think this type of thing should be planned: bam, bam bam, I've had enough of that. (Pn 4)

Another important aspect of the patients' motivation for doing YG arm training related to the nature of competition and benchmarking within the games. Both the women and men seemed to benefit from the YG applications that included incentives based on competition and rewards, because these factors made the training enjoyable and made them strive for more progress, as reported by the patients:

But I could see up in the corner, where it said how many points I had got… So I really wanted to get more. (Pn 4)

This reward system seemed to increase the number of repetitions. Furthermore, it increased the focus and concentration on the patient's abilities rather than on the impaired arm. Even those patients who had some cognitive impairments or were unacquainted with the system of counting points did enjoy the visual (points) potential progress they could make, and to compete with themselves, as confirmed in the following quotation:

I played against myself and enjoyed that, like, to improve myself. I could see, of course, that I scored 84%, but I found it hard to figure out what it was that gave points … the rules of the game?? Uh! What is it that gives points, what is it I have to go after, and also whatever gives the best training result. (Pn 2)

Another important dimension was the way the reward system of the different games challenged the patients to go for more and had a positive impact on the patients' moods, as described by a male patient while he explained how he enjoyed playing the most advanced games:

Then, suddenly, the eggs appeared, and they gave points. So I got five eggs in a row there. Well, I was high for the rest of the day. (Pn 3)

Furthermore, he appreciated in particular the progression of the game, which could be graded according to his abilities. He emphasized the therapists' skills and knowledge of the VR system, stating that, at any time, they could challenge the patient to do his very best.

The difficulty levels in YouGrabber, they were well hard. It wouldn't be right to say that you weren't challenged by it, because you could just increase the difficulty, and then it came automatically, you know? … And they (the therapists) were lightning fast, like, if you got over confident, you know? So they just increased it a couple of levels, so you had to stretch yourself again. (Pn 3)

All therapists also acknowledged and emphasized the motivational qualities of YG system.

I mean, for many it was motivating to work with virtual reality. That was obvious. (All therapists)

The therapists highlighted the reward system, benchmarking, and calculation of activity time.

You don't have to do that (count repetitions) in YG, it calculates all the percentages, doesn't it? (B) And counts points and calculates the activity … that's a good motivating factor. Yes, to reward work done so intensively sitting at a desk. That's important. (C)

However, in therapist opinion it did not seem to be for the purpose of giving joy; rather, it kept the patients in action.

Yes, in an awful lot of ways, in fact, so you could one way or another keep them a bit active. (G)

There were also a lot of them who said that they didn't notice at all that they did so many repetitions (A), because they were engrossed in looking at the screen and playing the game. (B)

…hat they get so wrapped up in, um, well, in the game that's going on, so they sometimes get a bit more movement than I would have anticipated. (D)

Based on the therapists' statements, it seems that the motivational factors were argued for by the fact that the YG system could increase the patient's activity, movements, and motivation in an almost manipulative way, so to speak.

They could see whether they had improved, and they could compete with themselves, both within the same training session and from day to day. (All therapists)

…they (patients) seemed more committed to this than to usual training. (C)

They (the patients) have that feeling that they themselves are making a difference in their training. (E)

From the therapists' perspective, the patients were more committed to the YG games than to usual therapy.

## 6. Engagement

The second theme to arise from the data relates to the patients' involvement. The theme also relates to the first theme, motivational factors. Both the patients and the therapists emphasized how the patients were engrossed in and occupied by the activity.

I was very surprised that I got so engaged in the task itself. (Pn 2)

I sort of threw myself into it, so I got to move my whole body, ha ha! (Pn 5)

But all in all it's because I get so damn engrossed in that stuff, you know? But I can get blisters from it, too, playing at something like that, you know? (Pn 3)

The patients described how they had to be attentive, alert, and ready for their next move:

Because you have to be alert the whole time and try to keep up with whatever comes next. And I think that was good. (Pn 4)

For some patients, this persistent involvement in the activity became a sort of exercise in improving their ability to concentrate. Achieving the limit of their fatigue was strenuous, but in the long run it had a positive outcome:

But then I've discovered that after half an hour I began to lose concentration. It's also a training in concentration. Uh, so, uh, I have become more and more aware of, one could say, when I've lost the thread. It also makes you aware that, hey, it was more because you couldn't concentrate than because your hand was bad. But I think in itself it helps with concentration just to sit for such a long time. It is very beneficial. (Pn 2)

It appeared, furthermore, that the patient gained more awareness of her capabilities and limits through this process.

One of the participating patients who had an itchy and unusual feeling in her paretic arm reported the following:

I felt, especially at the start, discomfort about that (points to her arm). I can't … I don't want to say actual pain, but discomfort in that bad arm. So, it has helped me just to forget the discomfort and train. So, I was surprised at that for a long time. (Pn 2)

This quotation descripted how YG training required full attention and might distract her from thinking about the strange feeling in her arm. She forgot about her discomfort by being occupied by the games.

## 7. Perceived Improvement

The main improvements inducted from the patients' interview data relate to a general use of the affected arm:

Well, you really got to use the arm, so it got better. (Pn 1)

It (the affected arm) has definitely got better. It's not working ok, you know. (Pn5)

However, some patients reported the improvements to be random and uncertain:

But it was really sort of a bit coincidental, what you could do (all depending on the games). (Pn 1)

I don't know how good it is. I can't say. I mean, I couldn't see any difference in the test before and after… So it didn't help an awful lot. (Pn 4)

Other patients highlighted training of specific functions, like fine motor skills and fingers and hand function:

It think it's been of benefit, especially in my fingers. (Pn 2)

But I'm sure it (the intervention) has contributed. Because this arm, it has improved faster than the legs, right? And I would nearly say that I can do everything, you know? Uh, except that it just spins a little in it, right? (Pn 3)

And I actually think that the fingers are some of the worst things to get moving. And it was that that shocked me most in therapy, where I thought, ah, these fingers, they're going to be the worst. So, in that way, I really think it has been a help… I mean, these fine motor skills, and I've now begun to knit, eh. (Pn 2)

The same patient also emphasized her improved ability to be focused and endure a task over a longer period.

However, a few patients mentioned that the training was too focused on the hand and that they missed situations where the hand, elbow, and shoulder were together involved in the movements.

The therapists, on the other hand, were more sceptical about patients' improvements, especially regarding the question of whether YG arm training could offer more than conventional arm training. However, they acknowledged the following:

But it could be that they can see that they can do several repetitions from one session to the next, and they can, and we can also measure this. So, that's an advantage. (A)

## 8. Individualization

The fourth theme concerned the YG system and the therapeutic pedagogical support to introduce the right game and level of game that would be suitable for each patient.

Yeah, right, we talked about that… toys, cars, carrots (preferred game), yes. And level of difficulty. And for how long? (Pn 1)

The therapists indicated that it is of the utmost importance that there is an opportunity to exercise the affected arm separately and thereby inhibit the unaffected arm:

Among the good characteristics of YG, which we've talked about in the group, is that the patients could actually avoid using the good hand. So, if they took over too much, you could just omit it from the game, so it was only the good hand they could use. But, whether they carried on after, we don't know. For example, those “magic fingers” (a type of mirror game). (F)

The therapists mentioned a number of factors that could adapt the training for individuals:

… set on each finger… different grips, graduate the game, time, tempo, etc. (All)

They also pointed out how easy it was within the system to increase the number of repetitions of each movement:

Well, the advantage is that there are an awful lot of repetitions. There were also several (patients) who said that they didn't notice that they had done so many repetitions, because they were engrossed in looking at the screen and playing the game. (E)

They get to do more than they otherwise would be able to manage, right. And they are more concentrated. (C)

The therapists also acknowledged that YG has a praise and reward system built in as a didactic tool. It is therefore both natural and easy to support the patient in doing more, giving them feedback and facilitating them in increasing their self-esteem.

The therapists also mention recognizability and security as factors that are important in order to achieve individualization:

And for some it was good, that, where they could recognize the games. That there were not more differences. Eh, there was something they knew, and so they were secure in it. That there computer thing, they were anxious beforehand. It was actually typical of the older ladies. (E)

They said, I can't get my head around it, when it comes to a computer. Yes, actually I can. “I can actually recognise it and now I know what it's all about.” (G)

## 9. Technological Issues

Patients expressed that the therapists' teaching, guidance, and support both at the introduction to the intervention and later in the subsequent VR training were of high quality. However, they did express frustrations with the device. This theme was expressed as an important issue, especially by the therapists and to some degree also by the patients.

There were significant emotional challenges for both patients and therapists when the screen and the machine were in action. The game could freeze. The goal was inaccurate (such as a carrot to be taken being slightly offset so the patient had to grab the shadow of the carrot). There were also some problems in completing games and in saving patient results. Some of the therapists found that the games' graphics were too primitive and that the games were too childish.

Despite the many frustrations regarding technical issues, all the therapists did recommend VR training as a significant supplement to the daily arm training for patients in the subacute phase. Furthermore, they argued that most patients could use it periodically as self-training or as part of team training, where the therapist supports the patient, if needed.

## 10. Overall Level of Satisfaction with VR Training

The questionnaire survey, addressing satisfaction with the VR training, was conducted among the health professionals and patients involved in the main study. Data from 45 patients were collected. The scale ranged from 1 (very satisfied/very much improved/very motivating) to 5 (not satisfied/not improved/not motivating). Most patients evaluated their satisfaction with VR training as “very satisfied” to “somewhat satisfied,” with a mean score of 1.5 (SD 0.75). Likewise, the perceived upper limb improvement during the VR training was rated by most patients as “much improved” to “very much improved,” mean score 1.6 (SD 1.0). Six patients (13%) reported only “slightly improved” or “not improved.” Many patients rated VR training as “motivating,” mean score 1.9 (SD 0.9). The free text remarks echoed comments obtained during the interviews. Patients commented on the perceived benefits, but also mentioned some technical difficulties.

A questionnaire with a similar range from 1 to 5 was handed out to therapists who carried out the VR training during the study. They were asked about the level of their general satisfaction in working with the VR system, the perceived ease of use, and their opinion on how motivating the VR training was for their patients. Overall, therapists were “somewhat satisfied,” mean score 2 (SD 0.6). The ease of use was rated 2.7 (SD 0.7), between “rather easy” and “neither easy nor difficult.” Predominantly, VR training was regarded as motivating for patients, rated between “motivating” and “somewhat motivating,” mean score of 2.4 (SD 0.8). Therapists also expressed some frustration about technical problems and the need for more games to increase variety.

## 11. Discussion of the Findings

The current qualitative substudy provides knowledge about patients' and health professionals' experiences of (and satisfaction with) using VR technology within subacute stroke arm training. Through semistructured face-to-face interviews with six patients, two focus group interviews with seven therapists, and 45 returned patient questionnaires and therapist questionnaires, various important experiences and views were highlighted. Six themes emerged: (i) motivational factors, (ii) engagement, (iii) perceived improvements, (iv) individualization of arm training, (v) frustrations about device malfunction, distresses, and concerns, and (vi) overall level of satisfaction with VR training.

Figure 2 illustrates the synthesis of the findings and discussion. The oval background illustrates playing and gaming as an underlying premise that affects motivation and improvement. The bold arrows illustrate themes that are expected to lead to increased motivation and improvement.

Both therapist and patient participants reported several themes of using the YG VR system. The most important theme seemed to be that using a VR device had been enjoyable. The therapist enjoyed seeing the patients activity and engagement and the patients enjoyed working with the YG system. Words such as playful, diversion, competing with oneself, surprising, and challenging were used by the patients. The nature of playing and characteristics of reporting points, giving level of activity as a percentage, and rewards appeared to have a significant influence on the patients' mood and level of engagement and seemed to promote a “gung-ho” spirit, in that they felt that they could perform more repetitions. The data analysis relates to James Carse's theories of finite and infinite games [19]. In the present VR system (YG), the games are structured as finite games with the purpose of winning. “If a finite game is to be won by someone it must come to a definitive end” [19]. This VR system seemed to be designed in such a way that the patients had to challenge themselves and thereby overcome more repetitions. YG has rules of play and uses temporal boundaries system (i.e., it has a clear beginning and a definitive, marked area), specified players, and a ranking at the conclusion of the play. So, even if VR is unfamiliar to participants and could initially promote a sceptical attitude, especially among some of the older participants, the games in this VR device have a recognizable structure, just like many well-known board games, which most people know from their childhood or adulthood.

Nevertheless, to some degree the YG games include elements of infinite games in that one of their purposes is to keep the participant continuing to train: “The only purpose of the infinite game is to prevent it from coming to an end; to keep everyone in play” [19]. Although an individual game can be completed, it seems that the patient will try again to score even higher points or want to try a similar but more challenging game. The game has a simulated “drug-like” effect on the participants: “I want more.”

Benchmarking has been mentioned in a previous study as an essential feature when dealing with training and learning [24]. Both patients and professionals described the significance of being able to measure oneself (e.g., one's progress or relapse) in conducting the VR intervention. An early study with both male and female respondents touches upon benchmarking [25]. The study describes how patients measure the parts of the body affected in relation to various standards in order to gain a sense of how far they have progressed in their training. In Gubrium et al.'s study [24], the respondents describe how they themselves assess progress by taking measurements of everyday functions. They assess who they are as people and their status seen in relation to the time before their stroke, to their own age, and to tasks in everyday life. This self-assessment, according to the participants, has considerable significance in respect of the participants' efforts for progress and is an important parameter for determining the effect of treatment. We do see a similar effect in the current study. The structure of the game as well as the didactic framework of individualization used by the therapists facilitated these aspects and seemed to increase the motivation of the patients to do more.

In the subacute phase, bodily changes appear to dominate as an issue. The attention is directed toward the way the body malfunctions and to find new ways of coping with everyday actions. In a Norwegian study, stroke individuals described how they gradually came to feel more confident with a body that they could no longer take for granted [26]. The body (and affected extremity) can become separated from the self and take on the form of an object (passive and foreign), inflicting a stage of nonuse, or it can be perceived as a stranger who does not “belong to or is not noticed” [26]. Therefore, it is important to focus on body image and body schema [27] in therapeutic interventions to prevent and minimise such conditions. Specifically, the concepts of body schema, body image, sense of ownership, and sense of agency [28] help in the differentiation/interpretation/understanding of signs and symptoms, bodily expressions, and verbal communication and thereby support intervention [29]. The body schema is a close-to-automatic system of processes that constantly regulate posture and movement to serve intentional action [28]. The current intervention and the design of YG make use of the concept of body schema by demanding adjusted finger, hand, and arm movements in order to solve the task presented on the screen. Furthermore, the YG device supports the concept of body image by visualizing the participant's own movement on a screen. The participants in the current study do have both sensory and motor impairments that disturb their body schema, their intentional actions, and the way they experience their bodies and the environments. Such concepts and issues should always be evaluated and considered in the therapist's clinical decision-making process and choice of intervention and didactics.

However, the analysis also pointed to patients' and therapists'* demotivation*. Device malfunctions seemed to be an annoying factor that was linked to a decrease of motivation. These problems have previously been described in several studies [11, 12].

Improved interprofessional cooperation and knowledge exchange between engineers of computer-based devices and therapists, as experts of human movement and patient-centred needs in daily living, could reduce the gap in end-user satisfaction [11, 12] left by the technology that is currently available. The collaboration desire is also supported by findings from Tatla et al. [30].

*Limitations and Strengths. *The scientific trustworthiness of the study was evaluated using the concepts of transferability, credibility, dependability, and confirmability [23, 31, 32]. To increase the credibility of the study, methods of triangulation were used.

The limited sampling of the current study and excluding patients with limited speech ability reduced the transferability to all subacute stroke patients, even though another study did find similar results in the case of chronic stroke. However, the participants in the current study had an appropriate age range from 23 to 79, with a range of educational backgrounds and problems additional to reduced arm function. This strengthens the transferability of the findings in the study. The therapists' years of experiences also show a broad range, from 3 to 25 years.

The main strength of this study lies in the correlation between patients' and health professionals' satisfaction with the intervention and the data generated in the interview survey. Respondent validation performed during the interview process also strengthens the findings in this study.

## 12. Conclusion

Patients and therapists mainly valued the intensive and motivational character of the VR training.

The playful nature of the training appeared to have a significant influence on the patients' moods and engagement and seemed to promote a “gung-ho” spirit, so they felt that they could perform more repetitions. Technical challenges compromised the positive experience to a certain extent.

## Figures and Tables

**Table 1:** Sample characteristics, participating patients.

Participants	Gender	Age	Diagnosis/affected side	Days after stroke	ARAT^*∗*^ before intervention	ARAT after intervention
Patient number 1 ID 87	Female	79	Infarct/right	12	44	49
Patient number 2 ID 108	Female	55	Haemorrhage/right side	8	38	57
Patient number 3 ID 105	Male	54	Haemorrhage/right side	35	30	38
Patient number 4 ID 117	Male	73	Infarct/right side	8	26	39
Patient number 5 ID 120	Female	68	Infarct/right side	11	37	38
Patient number 6 ID 79	Female	33	Haemorrhage/Right	69	41	48

^*∗*^Action Research Arm Test (ARAT).

**Table 2:** Sample characteristics of the health professionals.

Professional experts	Gender	Years of experience as a health professional	Years of experience in neurorehabilitation	Number of courses of YouGrabber conducted	Experienced in other VR systems	Focus group interview
Specialist occupational therapist, A	Female	6	3	7	No	F1
Physiotherapist, B	Female	17	12	7	No	F1
Physiotherapist, C	Female	25	12	4	No	F1
Occupational therapist, D	Female	13	9	18	Yes	F2
Occupational therapist, E	Female	18	5	4	Yes	F2
Physiotherapist, F	Female	3	2	18	Yes	F2
Physiotherapist, G	Female	9	8	18	Yes	F2

**Table 3:** Interview guide.

Main categories	Questions
(1) General overall impression of YG training	Can you tell me what made you say yes to joining the YG project? What is your overall impression of YG training? Can you describe some good qualities of YG? Can you describe some negative qualities of YG? Which games do you prefer? Which games did you not like?

(2) Experiences of other rehabilitation technology	Had you tried other technological rehabilitation equipment? How did you like it? Would you like to try other technological rehabilitation equipment?

(3) Experiences of progress in YG training	Did you experience progress with your arm function related to YG training? Did you experience discomfort, or annoyance, that you think was caused by YG training?

(4) The therapists' learning strategies, support, organization, and facilitation of the YG training	Can you describe how the start-up with YG was? Was it hard to learn, to use, to understand at the outset? Can you describe how you experienced YG at the end of the course? Was the 4-week timeframe an appropriate timeframe? How did you experience the length of the session/processing time (up to 60 minutes)? How did you experience the therapists' teaching, guidance, support during the training? Do you have recommendations for the therapists using YG? Would you recommend YG training to your fellow patients?
